# Whole-Genome Sequence and Annotation of *Salmonella enterica* subsp. *enterica* Serovar Enteritidis Phage Type 8 Strain EN1660

**DOI:** 10.1128/genomeA.01517-16

**Published:** 2017-01-26

**Authors:** Benjamin J. Perry, Stephen F. Fitzgerald, Carsten Kröger, Andrew D. S. Cameron

**Affiliations:** a Department of Biology, University of Regina, Regina, Saskatchewan, Canada; b Department of Microbiology, School of Genetics and Microbiology, Moyne Institute of Preventive Medicine, Trinity College Dublin, Dublin, Ireland

## Abstract

The genome of *Salmonella enterica* subspecies *enterica* serovar Enteritidis phage type 8 strain EN1660, isolated from an outbreak in Thunder Bay, Canada, was sequenced to 46-fold coverage using an Illumina MiSeq with 300-bp paired-end sequencing chemistry to produce 28 contigs with an *N*_50_ value of 490,721 bp.

## GENOME ANNOUNCEMENT

The bacterial pathogen *Salmonella enterica* presents a significant health burden globally and remains a persistent challenge in North America, particularly for the agriculture and food production industries that work diligently to prevent *Salmonella* contamination. Nontyphoidal *Salmonella* is estimated to cost the U.S. economy $3.7 billion per year due to hospitalizations and premature deaths (1). *Salmonella enterica* subsp. *enterica* serovar Enteritidis is the most common clinical isolate in the United States, yet Salmonella Enteritidis genomes are under-represented in DNA sequence databases. Here, we report the whole-genome sequencing of Salmonella Enteritidis strain EN1660, a clinical isolate from a 1992 community outbreak in Thunder Bay, Ontario, Canada, associated with consumption of contaminated poultry products. EN1660 was classified as phage type 8 (PT8) by the Ontario Laboratory Centre for Disease Control and was determined to be motile, capable of forming biofilm, and highly invasive in Caco-2 cell culture (2). EN1660 is classified as *Salmonella* sequence type 11 (ST11) by MLST version 1.8 (3).

Genomic DNA isolation was performed using a BioBasic Canada DNA isolation kit. DNA sequencing libraries were prepared using the NEBNext Ultra DNA library prep kit, with a final library size selection of 700 bp. Template sequencing was performed using an Illumina MiSeq and V3 300-bp paired-end sequencing chemistry. Demultiplexed sequencing data were filtered for phiX sequences using Bowtie2 (4) and the *Enterobacteria* phage phiX174 *sensu lato* reference sequence (RefSeq: NC_001422.1) for comparison. Filtered sequencing reads were then quality trimmed using Trimmomatic (5), removing GC% skews from the beginnings and ends of the reads and trimming at an average *Q* score below 20 in a sliding window of 5 bp. This resulted in 985,195 high-quality paired-end reads for use in genome assembly.

The genome was assembled using “careful” mode with a *k*-mer size of 127 in SPAdes version 1.35 (6). The resulting assembly had a consensus length of 4,701,324 bp spanning 28 contigs, with an *N*_50_ value of 490,721 bp and an *L*_75_ value of five contigs. The average genome coverage was 46× with a GC % of 52.1%. Annotation of the draft genome sequence was conducted using the NCBI Prokaryotic Genome Annotation Pipeline (7). Genome annotation predicted 4,478 coding sequences, eight rRNA operons, 78 tRNAs, 17 ncRNAs, and two CRISPR arrays.

The EN1660 draft genome was screened for antibiotic resistance genes using the Antibiotic Resistance Gene Database (8), which predicted the presence of four resistance gene homologs: *mdtM*, *pbp*2, *acrB*, and *mdtL*. The draft genome sequence was also analyzed for prophage content using the PHAge Search Tool server (9). PHAST analysis revealed six prophage regions, of which two are intact (similar to Gifsy-2 and RE-2010); one more is potentially fully intact (similar to *Pseudomonas* phage B3); and three are incomplete. We used BLAST to confirm the presence of the broadly distributed *Salmonella* pathogenicity islands SPI-1, SPI-2, SPI-3, SPI-4, SPI-5, SPI-6, SPI-9, SPI-11, SPI-12, SPI-13, SPI-14, and SPI-16 in strain EN1660.

### Accession number(s).

The draft whole-genome sequence for *Salmonella enterica* subsp. *enterica* serovar Enteritidis EN1660 was deposited into the NCBI GenBank database under the accession number LUUA00000000.
